# Sediment as a Potential Pool for Lipophilic Marine Phycotoxins with the Case Study of Daya Bay of China

**DOI:** 10.3390/md17110623

**Published:** 2019-10-31

**Authors:** Yang Liu, Peng Zhang, Sen Du, Zhuoru Lin, Yanyan Zhou, Lizhao Chen, Rencheng Yu, Li Zhang

**Affiliations:** 1 Key Laboratory of Tropical Marine Bio-resources and Ecology, Guangdong Provincial Key Laboratory of Applied Marine Biology, South China Sea Institute of Oceanology, Chinese Academy of Sciences, Guangzhou 510301, China; liuyang@scsio.ac.cn (Y.L.); zhangpeng_nanjing@126.com (P.Z.); dusen14@mails.ucas.ac.cn (S.D.); zhouyyahgy@scsio.ac.cn (Y.Z.); chenlizhao13@mails.ucas.ac.cn (L.C.); 2 Laboratory for Marine Ecology and Environmental Science, Qingdao National Laboratory for Marine Science and Technology, Qingdao 266071, China; linzhuoru@outlook.com; 3 Southern Marine Science and Engineering Guangdong Laboratory (Guangzhou), Guangzhou 510301, China; 4 Institution of South China Sea Ecology and Environmental Engineering, Chinese Academy of Sciences, Guangzhou 510301, China; 5 University of Chinese Academy of Sciences, Beijing 100039, China

**Keywords:** lipophilic marine phycotoxins, sediment, liquid chromatography-tandem mass spectrometry, Daya Bay, toxin composition, spatial distribution

## Abstract

Marine sediments can reserve many environmental pollutants. Lipophilic marine phycotoxins (LMPs) are natural toxic substances widespread in the marine environment; however, evidence of their existence in sediment is scarce. In the present study, in order to explore the occurrence and distribution characteristics of LMPs in sediment, surface sediment samples collected from a tropical area of Daya Bay (DYB) at different seasons, were analyzed using liquid chromatography with tandem mass spectrometry (LC-MS/MS). According to the results, up to six toxin compounds were detected in sediment samples from DYB, OA and DTX1 had the highest levels, followed by PTX2, homo-YTX, AZA2, and GYM. Although AZA2 and GYM were found in most of the sediment, OA, DTX1, homo-YTX, and PTX2 were the predominant toxin compounds, and PTX2 was the most ubiquitous toxin in sediment. The spatial distribution of LMP components in the sediment fluctuated with sampling times, partially according to the physical–chemical parameters of the sediment. There are likely several sources for LMPs existing in surface sediments, but it is difficult to determine contributions of a specific toxin-source in the sediment. Therefore, marine sediments may be a toxin reservoir for LMPs accumulation in benthic organisms via food chains.

## 1. Introduction

Lipophilic marine phycotoxins (LMPs) are natural organic pollutants produced by some species of marine microorganisms [1]. LMPs can be easily accumulated in mollusks and are considered to be emergent toxicants affecting food safety in the shellfish food industry. The joint Food and Agriculture Organization (FAO), (Intergovernmental Oceanographic Commission (IOC), and World Health Organization (WHO) reports propose that LMPs can be classified into these groups, namely, okadaic acid (OA) and dinophysistoxins (DTXs), pectenotoxins (PTXs), yessotoxins (YTXs), azaspiracids (AZAs), cyclic imines like 13-desmethyl spirolide C (desMeC) and gymnodimines (GYMs) [1]. With the ever-increasing contamination and toxic effects on human health caused by LMPs, these toxins have been attached many concerns worldwide. Potential hazardous properties of LMPs have been taken into consideration, and efforts have been made to understand the contamination status of LMPs in the marine environment.

Currently, extensive information for toxins in shellfish, toxic microalgae or phytoplankton has been provided in previous studies [2,3,4,5]. Generally, intracellular toxins in algae are regarded as a source of toxin accumulation in filter-feeding marine animals. Nevertheless, extracellular LMPs also exist in marine environments after toxigenic algae cells declined [6]. LMPs, as hydrophobic organic compounds (HOCs), have strong affinity for natural sorbents, such as suspended particle matter (SPM) [7], and can sink from the surface water to the benthos by sedimentation [8]. The marine sediment is an important source of hydrophobic pollutants, such as sterol dinosterol [2,9,10]. It has been found that the marine sediment has the capacity to reserve LMPs in the area toxin-producing algae widely distributed. Hitchcock et al. showed that the liposoluble constituent BTX group toxins could be detected in marine sediments from the coast of Florida, USA [11]. In China, some LMP components were also found in the sediment samples from the East China Sea (ECS), Bohai Sea (BS), and Yellow Sea (YS) [12,13]. 

Daya Bay (DYB) is one of the largest and most important gulfs along the coast of the Southern China Sea (SCS) (22°30′–22°50′, 114°30′–114°50′) with shallow water (depth 6–15 m) [14]. As a semi-enclosed inland sea, DYB is an important spawning and breeding ground for many marine organisms, especially some benthic shellfish are consumed by the locals and sold to the public [15]. Recently, anthropogenic activities such as petrochemical enterprises, marine transportation, nuclear power plants and marine aquaculture industry have significant impacts on the aquatic environment of the DYB [15,16]. The nutrient level and the eutrophication status have increased in this area with economic development, and the abundance and proportion of toxigenic algae-producing LMPs are also increased in the waters of DYB [15,17]. Fisher et al. reported that HOCs could be absorbed on sediment and caused acute toxicity to aquatic organisms [18], and sediment could increase OA and DTX1 accumulation by mussels [19]. Given the hydrophobic property of LMPs, marine sediment was considered to be the final sink of hydrophobic organic contaminants. However, reports related to LMPs in the sediment of this area are very scarce.

The goal of this study was to assess the distribution characteristics of LMPs in the sediment of DYB, and we did a pilot study for the fate of LMPs in the marine environment. Sediment samples obtained from consecutive research cruises in the subtropical area of DYB located in the SCS, were analyzed by targeted HPLC-MS/MS. The distribution characteristics of LMP concentration and composition in surface sediment samples from DYB will offer a sound basis for predicting the possible risk to marine benthic shellfish.

## 2. Results and Discussion

### 2.1. Occurrence of LMPs in the Sediment of DYB

**Figure 1:**
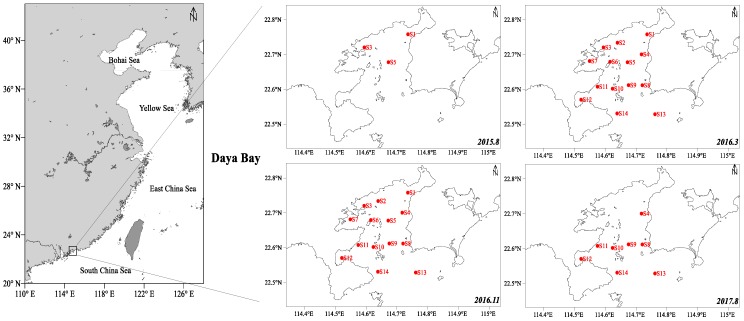
Sampling location of surficial sediment samples in the sea area of Daya Bay (sampling time: August 2015, March 2016, November 2016, and August 2017)

**Figure 2:**
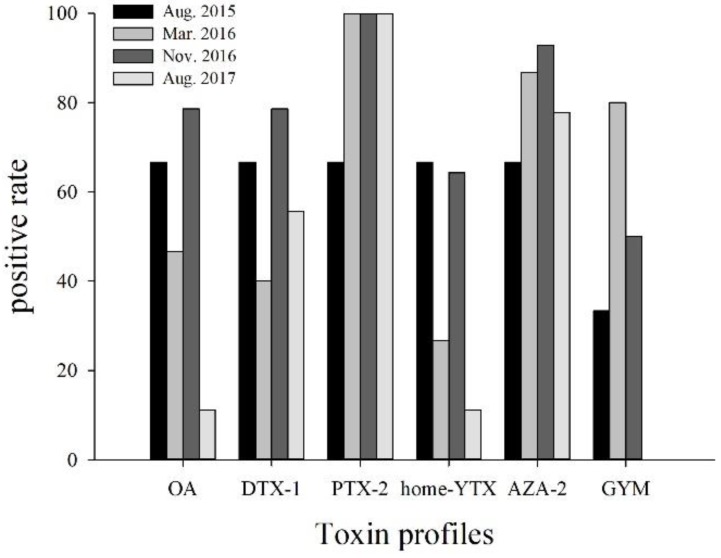
Positive rate of multiple lipophilic marine phycotoxins detected in surface sediment of Daya Bay at four different sampling times.

**Figure 3:**
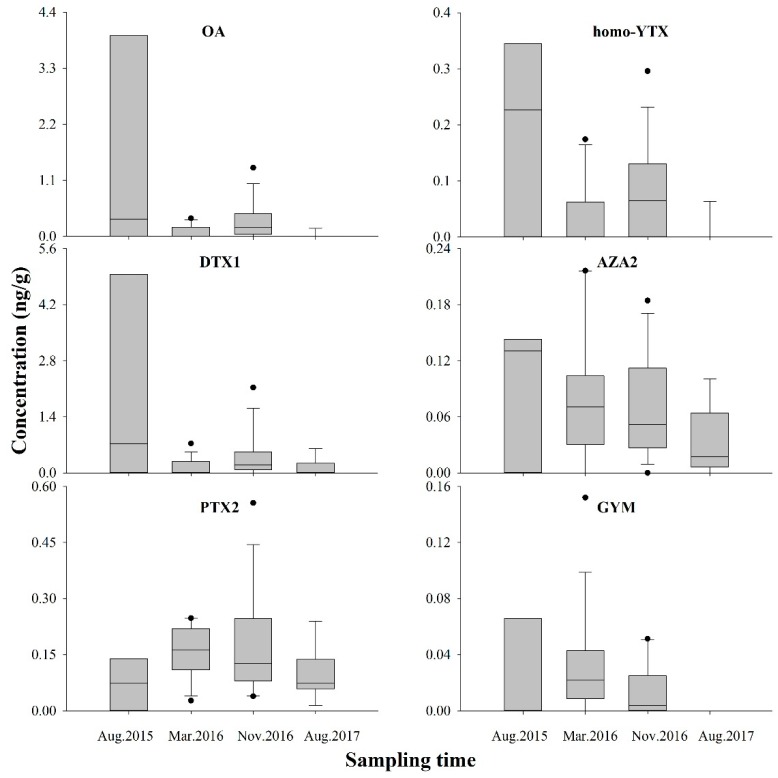
Concentrations of multiple lipophilic marine phycotoxins detected in surface sediment of Daya Bay at four different sampling time (ng g^−1^ dry weight).

Data of LMPs in the area of DYB is very limited, and LMPs in oysters [17,20] and phytoplankton samples [17] from one aquaculture site in Dapeng Cove are the only reports at present. For the first time, this study presented the composition and distribution of LMP in the surface sediment of DYB (Figure 1 and Table S1 in the Supplementary Materials). As shown in Figure 2; Figure 3, up to six toxin compounds were detected in sediments, including OA, DTX1, PTX2, homo-YTX, AZA2, and GYM. OA, DTX1, and PTX2 were the most abundant LMP components in sediments of DYB. In China, OA, DTX, and PTX2 were the only toxin compounds found in sediment samples collected from Hangzhou Bay of the ECS, Laizhou Bay of the BS and Jiaozhou Bay of the YS [12,13], which was consistent with other studies and previous observations in seawater and phytoplankton [13,14]. 

The existence LMPs in sediments was different with toxin profiles. PTX2 tended toward the highest positive rate, ranging from 66.7–100.0%, followed by AZA2 (66.7–92.9%), GYM (0.0–80.0%), DTX1 (40.0–78.6%), OA (11.1–78.6%), and homo-YTX (11.1–66.7%) (Figure 2), indicating that PTX2 was the most ubiquitous toxin in sediment, similar to the reports in the Baltic Sea [8]. Compared to PTX2, although OA, DTX1, and homo-YTX had lower detection rates, they were in higher levels; this may be caused by higher sedimentation rates of OA and DTX1, and rapid elimination of PTX2 [8]. According to the results, OA and DTX1 had the highest levels ranging from 95.5–3937.8 and 108.1–4958.2 pg g^−1^ respectively, followed by PTX2 14.2–273.8 pg g^−1^, homo-YTX 60.0–350.0 pg g^−1^, AZA2 20.0–220.0 pg g^−1^, and GYM 10.0–150.0 pg g^−1^, similar to reports from Haizhou Bay of the YS, where OA and DTX1 concentrations were 330–4280 and 580–25,320 pg g^−1^, respectively [13]. OA was the predominant component in the sediment of Hangzhou Bay with 186.0–280.7 pg g^−1^ [12]. Generally, LMP in the sediment of DYB had lower concentrations but more complex composition than that in other reported sea areas.

### 2.2. Spatial Distribution of LMPs in the Sediment of DYB

**Figure 4:**
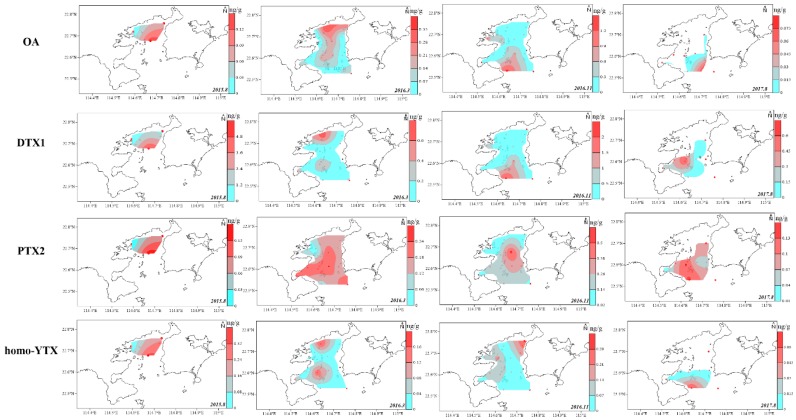
The spatial distribution of the predominated lipophilic marine phycotoxins okadaic acid (OA), dinophysistoxin (DTX)1, pectenotoxin (PTX)2 and homo-yessotoxin (YTX) detected in the surface sediment of Daya Bay at different sampling times from 2015 to 2017 (ng g^−1^ dry weight).

**Figure 5:**
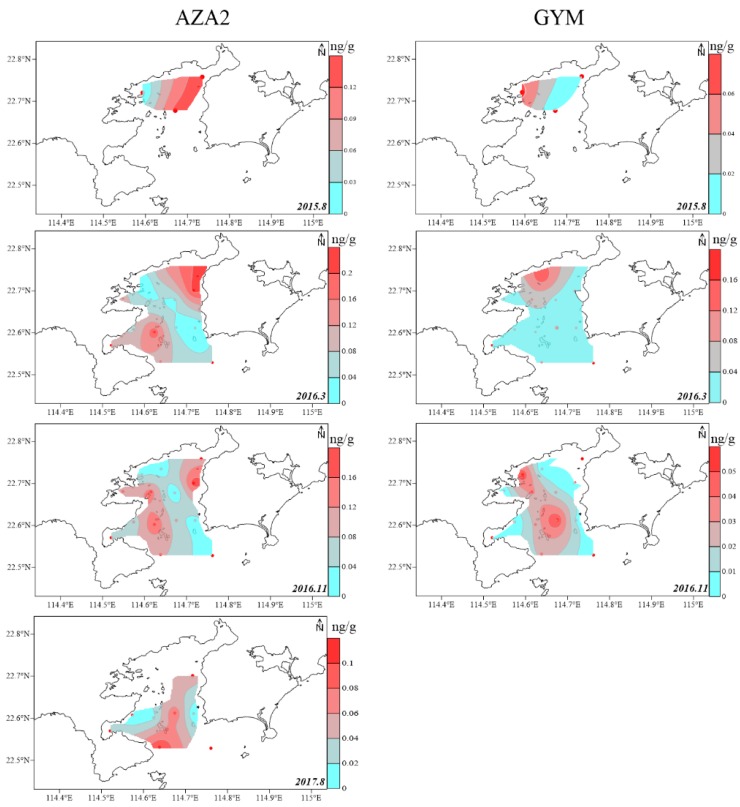
The spatial distribution of the predominated lipophilic marine phycotoxins azaspiracid (AZA2) and gymnodimine (GYM) detected in the surface sediment of Daya Bay at different sampling times from 2015 to 2017 (ng g^−1^ dry weight).

The spatial LMP distribution in the surface sediment was limited by marine sediment sites and was site-specific (Figure 4 and Figure 5). The spatial distribution of toxin components, in the surface sediment of DYB were shown in the diagrams of Figure 4 and Figure 5, and no GYM was detected in sediments from August 2017 (Figure 5). Generally, PTXs and OA were always concurrent in the marine environment, and their spatial distribution was highly consistent [20], which is mainly because they originated from *Dinophysis*, simultaneously [3,12]. The spatial distribution of these predominant phycotoxins in the sediment of DYB fluctuated with sampling times (Figure 4). In August 2015, the limited sampling sites did not allow a good interpretation of LMP distribution in DYB, however, only OA and DTX1 occurred in S5 (114.67° E, 22.68° N) located in the middle of the bay, at extremely high levels of 3.94 and 4.96 ng g^−1^ respectively, where red tide was prone to contemporaneous eruption (not published), and other toxins also mainly distributed in the middle of the bay. In March 2016, OA mainly distributed from the north shore to the mouth of the bay along the center of DYB; the distribution range of DTX1 was slightly narrower than OA, and mainly distributed near the shore in the northern offshore area of DYB. At all the sampling times, PTX2 had the largest distribution range among these toxin profiles, indicating that PTX2 was the most widely distributed in the sediment of DYB, similar to PTX2 with the more extensive distribution in phytoplankton from the YS and ECS (unpublished data). However, the spatial distribution of OA and DTX1 changed dramatically with time. In November 2016, the high-value zones mainly occurred in the mouth of the bay, and it changed to the area of water of the bay in August 2017. PTX2 basically covered the survey area during the corresponding period.

Toxin homo-YTX has never been reported in the sediment before this study, and its spatial distribution was quite different from the OA and PTX groups (Figure 4). In August 2015, the zone with high value of homo-YTX was consistent with OA, DTX1, and PTX2, mainly due to a harmful algae bloom event that occurred in S5 during this period. In March 2016, there were two apparent distribution areas of DYB, as one located in the middle of the bay, and the other located north of the bay. In November 2016 and August 2017, the spatial distribution of homo-YTX changed dramatically. In November, homo-YTX was mainly distributed in a western region with a relatively high level in S3 (0.12 ng g^−1^), S6 (0.16 ng g^−1^), and S11 (0.12 ng g^−1^), and the northeast coast of DYB with relatively high concentrations in S1 0.30 ng g^−1^ and S4 0.17 ng g^−1^. However, in August 2017, homo-YTX was only detected in S14, which is located in the southwest of DYB near the outer sea.

### 2.3. Potential Source of LMPs in Sediment of DYB

LMPs are ubiquitous environmental pollutants and can be introduced into the environment via various routes. The sedimentation of toxin-producing algae cells, SPM absorbing toxigenic algae, dissolved toxins in seawater, and fecal pellets of zooplankter, are likely to be the possible sources of LMPs in sediments [8,21], but it is difficult to determine the extraction contributions of a specific toxin-source in the sediment. Due to the high hydrophobicity of lipophilic compounds and adsorption capacity of SPM and sediment, dissolved LMPs, toxigenic algae cells tend to be absorbed onto organic fractions of sediments and SPM in aquatic environment [7,22]. Recently, as a consequence of human activities in DYB, such as aquaculture and sewage discharge, toxigenic algae production and the abundances of SPM have increased, and contributes to the possibility of LMPs in the sediment. 

The complexity of LMP compositions in the sediment of DYB indicates the biodiversity of toxigenic algae producing LMPs in this area (Table 1). Generally, OA, DTX1, and PTX2 are considered as the most frequent components of LMPs, with high levels in phytoplankton [4], seawater [6,23], SPM [8], and sediment [12]. In this study, OA, DTX1, and PTX2 were the main LMP components in the sediment of the DYB. PTXs and OA were always concurrent in the marine environment and their spatial distribution is highly consistent [20], because they were mainly originated from *Dinophysis* simultaneously [3,12]. Phytoplankton is high abundant in the waters of DYB, including toxigenic algae such as *Dinophysis* species (*D. caudata* and *D. acuminata*) producing OA and PTXs [17], *Prorocentrum lima* producing OA. 

The YTX toxin components have not been reported in the surface sediment [8,12,13], while they have been extensively reported in YTX causative algae [23], phytoplankton [4], and shellfish [5]. Dinoflagellates *Lingulodinium polyedrum*, *Protoceratium reticulatum*, and *Gonyaulax spinifera* have been identified as producers of the YTX group [23] in the coastal waters of China. Although vegetative cells of YTX-producing algae have not been identified in the area of DYB, *P. reticulatum* cysts were widely distributed in the surface sediment of this area [16]. In this study, homo-YTX was the only component of the YTX group with the high level, and some strains of *P. reticulatum* only produce homo-YTX [24], therefore, homo-YTX in sediment might be produced by *P. reticulatum* that existed in DYB. 

AZA2 and GYM have also been putatively detected in shellfish and phytoplankton in China [4,5], and GYM was widespread in shellfish of the SCS compared to other areas [3,20]. However, AZA2 and GYM have never been reported in marine sediment until this study. In the Asia-Pacific region, *Azadinium poporum* is the causative producer of AZAs with AZA2 being the predominant compounds [25]. In the BS and SCS, *A. poporum* was responsible for AZA2 and two AZA-related compounds in phytoplankton and seawater [4,23,26]. Therefore, the presence of AZA-2 in the sediment of DYB might arise from *A. poporum*. The dinoflagellates *Alexandrium ostenfeldii* and *Karenia selliformis* were capable of producing GYM [27]. Currently, *K. selliformis* has not been identified in the coastal waters of China [4,5]. Although *A. ostenfeldii* was always considered to be a background species with low density, and it had been reported in the SCS and BS, and resting cysts produced by *A. ostenfeldii* were common in the surface sediment [28]. Therefore, *A. ostenfeldii* may be the main producer of GYM in the sediment of DYB.

### 2.4. Relationship Between Physicochemical Property of Sediment and Distribution of LMPs in the Sediment of DYB

The fate of LMPs in the marine environment is poorly understood so far. According to the results described above, the incorporation of LMPs into underlying sediments may potentially be a long-term sink. Sedimentation of LMPs and physicochemical property of sediment, such as grain size, total organic carbon (TOC) and total nitrogen (TN), possibly influence the existence of LMPs in sedimentary environments [8,22]. Therefore, it is necessary to determine the occurrence of LMPs in relationship to sediments. 

The grain size of the sediments collected from DYB demonstrated the sediments were composed largely by clay (28.2 ± 1.4%), silt (58.2 ± 1.9%) and sand (12.4 ± 2.6%) (Table S2 in the Supplementary Materials). The average grain size of sediment was 4.82–7.72 and the median diameter was 4.11–7.61, without significant differences at different sampling sites and times (*p* > 0.05). These results indicated that surface sediments of DYB were mainly of a fine-grain silt and clay composition, supporting the ability of sediment to retain LMPs. Sediment grain size may be an important factor in regulating the sedimentation, adsorption, and distribution of some pollutants [32], and the viscosity and adsorbability of the marine sediment increase as the particle size decreased [32]. However, LMP concentrations also showed no relationship with the average grain and median particle diameter of sediment (Figure S1 in the Supplementary Materials). It was demonstrated that the diversity and richness of cysts in cohesive sediment are much higher than that in sandy sediment [33], and lipophilic brevetoxins flocculate easily with clays [34]. The high proportion of clay and silt makes LMPs and cysts to be easily retained in the sediment. 

Organic matter (OM) is the main part of sediment, which is prone to be accumulated and preserved in fine-grained sediments [35]. Kuuppo et al. thought that OA and DTXs were likely sedimentation in the OM component of sediment, such as fecal pellets [8]. TOC can control the levels of polycyclic aromatic hydrocarbons (PAHs) and LMPs in the sediment, because the viscosity and adsorbability of sediment increase with increased TOC content [32,36]. In this study, the measured TOC ranged from 4–25% with an average of 14%, and TN was 0.40–1.96 with an average of 1.06, the calculated TOC/TN varied from 5.14–13.06 with a mean of 7.91, and the total toxin concentration did not show a significant correlation with TOC and TN in the sediment. This may indicate no discrepancy in TOC and TN in the sediment of DYB (Table S3 in the Supplementary Materials). Notably, due to continuous input of fresh OM on the surface seafloor sediment [22], the sediment of DYB showed a strong reducibility with redox potential (Eh) oscillating anoxic conditions (−319 to −87 mV), and reducibility of sediments and stable toxic condition could promote the growth of reducing bacteria and the degradation of OM [37]. Therefore, in the present study, the concentration of total toxins had a significant negative correlation with Eh (*p* < 0.05) (Figure S1 in the Supplementary Materials), indicating that the sediment of DYB was not conducive to the preservation of LMPs. This might explain why the LMP concentrations detected in the sediment of DYB were not significantly different between the season surveys, and lower than reported in sediments from other sea area.

In this study, LMP concentrations in August 2015 and 2017 were much lower than that in March and November 2016. The positive rate of LMPs in sediments also showed the same trend, with the highest in November 2016, followed by March 2016, and lowest in August of 2015 and 2017. This indicated that LMPs in the sediment were spread more widely in the cold months (November and March) than in the warm months (August). Temperature may be an important factor affecting the adsorption of LMPs by sediments. Meanwhile, the temperature of November and March in this area was more favorable to the algal proliferation [4,14], and it might also be a reason for high toxin levels in sediments during these periods. Previous study had shown that temperature had a greater effect on HOCs concentrations in sediment [32], which could be explained by the water solubility, therefore, the negative correlations between LMPs incorporated in underlying sediments and the temperature are shown in results of this study. Because the sediment temperature was difficult to determine, the measured real-time temperature of the bottom seawater was used to indicate the sediment temperature. The average water temperature in the coastal region of DYB was 29.3 °C in the summer (July to September) and 17.3 °C in the winter (December to February) [37], similar to the temperature recorded in the present study.

## 3. Experimental Section

### 3.1. Chemical and Reagents

Analytical grade chemicals and HPLC-grade solvents were used in the study. HPLC-grade solvents, ammonium formate was purchased from Sigma-Aldrich (28%, Steinheim, Germany), acetonitrile and methanol were purchased from Merck (Darmstadt, Germany). The 18.2 ΜΩ cm^−1^ water was prepared by the Milli-Q water purification system (Millipore Ltd., Bedford, MA, USA) to configure LC mobile phases.

Certified reference materials (CRM) including okadaic acid (OA), dinophysistoxins-1 (DTX-1), pectenotoxins-2 (PTX-2), yessotoxin (YTX), homo-yessotoxin (homo-YTX), gymnodimine (GYM), azaspiracid-1 (AZA-1), azaspiracid-2 (AZA-2), azaspiracid-3 (AZA-3) and 13-desmethyl spirolide C (desMeC) were purchased from National Research Council-Institute for Marine Biosciences (Halifax, NS, Canada). The Standard toxins were dissolved in HPLC-grade methanol (Darmstadt, Germany) at the concentrations of OA (141.4 ng mL^−1^), DTX1 (166.0 ng mL^−1^), PTX2 (50.3 ng mL^−1^), YTX (63.9 ng mL^−1^), homo-YTX (116.0 ng mL^−1^), AZA1 (63.9 ng mL^−1^), AZA2 (20.8 ng mL^−1^), AZA3 (24.4 ng mL^−1^), GYM (29.8 ng mL^−1^), and desMeC (91.0 ng mL^−1^) as the mixed calibration solutions for later use.

### 3.2. Investigated Area and Sampling Collection

Surface sediment samples were collected in four targeted cruises carried out on August 2015, March 2016, November 2016, and August 2017 in DYB (Figure 1). Approximately, 250 g samples of surface sediment (top 0–10 cm) were collected from 14 sampling sites using a grab-box sampler, and one sediment sample was collected at per sampling site. All the marine sediment samples were preserved in a hermetic bag and stored in a refrigerator at −20 °C until treatment.

### 3.3. Sample Pre-Treatment and Toxin Extraction

Before analyzing physicochemical property and extracting toxins of sediment, sediments were first homogenized and freeze-dried with a vacuum freeze dryer. The sediments were freeze-dried, grinding crushed and sieved using a 2 mm mesh and stored in a tube in −20 °C for future treatments for determination physicochemical parameters of sediments and extraction LMPs in sediments.

First, accurately weighting the freeze-dried, crushed and homogenized sediment samples, and carbonates in sediments were removed with acidification using the 10% hydrochloric acid for 24 h. Acidified sediment samples were rinsed with distilled water and dried, TOC and TN were analyzed using a PE 2400 Series II elemental analyzer (Perkin-Elmer, Waltham, MA, USA).

Second, accurately weighting the pre-treated sediment samples, and the granulometry of the sediments was analyzed using a Malvern Mastersizer 2000 laser diffractometer that was capable of analyzing particle sizes between 0.02 and 2000 µm. Three groups of grain size >63 µm (sand), 4–63 µm (silt) and <4 µm (clay) were determined as percentages. 

Third, the extraction of LMPs from sediment samples was done by sonication with methanol (MeOH). 10 g aliquot of dried sediment sample were weighed and ground, then transferred into 15 mL centrifuge tubes, and 3 mL MeOH was added. The sediment samples with MeOH were vortex-mixed for 1 min using a vortex mixer (IKA, Staufen, Germany). Ultrasonic extraction was undertaken for 10 min, and samples were then centrifuged. The supernatant was transferred to a 10 mL volumetric flask and the pellet was re-extracted. Following the re-extractions, the supernatants were combined, and the final volume was made up to 10 mL. 

A clean-up and enrichment method for methanolic extracts of sediment involving solid phase extraction (SPE) has been developed [4,5]. Strata-X cartridges (3 cm^3^ 60 mg^−1^, Phenomenex, Milford, MA, USA) were used to remove matrix and enriched toxins according to the following procedure. The SPE cartridge was activated and equilibrated twice with 3 mL MeOH and 3 mL deionized water, successively. Before the samples were loaded on the cartridge, an accurate measured methanolic extract was diluted with water to 30% (*v*/*v*) MeOH/water solution. To remove some polar substances, 20% (*v*/*v*) MeOH/water solution was used to wash the cartridge, and the cartridge was dried under vacuum. Then the targeted toxins absorbed onto the cartridge were eluted with MeOH containing 0.3% (*v*/*v*) ammonium hydroxide, and the cartridge was dried under vacuum to collect residual MeOH. Prior to analysis, the eluent was mixed and filtered by a syringe nylon membrane (0.22 µm, Jinteng, China) prior to analysis with HPLC-MS/MS.

### 3.4. Analysis Method

LMPs in the sediment extractions were qualitatively and quantitatively analyzed using a previous method with modification [4,5]. Chromatographic separation of targeted toxin compounds was conducted by a Thermo Fisher UltiMate 3000 HPLC system with Waters X-Bridge C18. Mobile phase A was composed of acetonitrile/water (10:90, *v*/*v*) and 7 mmol L^−1^ ammonium hydroxide (pH 10.8); mobile phase B was composed of acetonitrile/water (90:10, *v*/*v*) and 7 mmol L^−1^ ammonium hydroxide (pH 10.8). Gradient program used in the analytical method was 10% B for 0–1.0 min, from 10% B to 90% B for 1.0–9.0 min, 90% B for 9.0–12.0 min, and 10% B for 12.0–18.0 min. The injection volume and flow rate were set as 2 μL and 0.4 mL min^−1^, respectively.

The mass spectrometer ABI-SCIEX-4500 Q-Trap (Applied Biosystems, Darmstadt, Germany) equipped with a TurboSpray^®^ interface was performed with mass spectrometric detection for target compounds. Both negative and positive electrospray ionization (ESI), and multiple reaction monitoring (MRM) mode, were operated in the mass spectrometer at the three different retention time windows. The first window was in negative mode and contained 10 transitions to determine OA, DTX1, DTX2, YTX, and homo-YTX. While both the second and third windows were in positive mode, each window contained 6 transitions, to determine AZA1−3, and SPX1, GYM, and PTX2, respectively. 

Qualitative and quantitative analysis of specific toxins were selected for each toxin with two parent/product ion pairs (Table 2). In addition, the ion scan range was set as 100–1300 m/z, the curtain gas (CG) was set as 35, the collision gas (CAD) was set as medium level. In positive and negative ionization modes, the ion spray voltage (ISV) was respectively set as 5500 (+) V or 4500 (−) V, and the ion source temperature was set as 500 °C. The concentration of LPs in sediment was expressed as ng g^−1^ dry weight (hereafter ng g^−1^ DW).

The chromatograms of HPLC-MS/MS for LMP standards and sediment samples were shown in Figure S2 in the Supplementary Materials, and the calibration curves of these toxins also presented in Figure S3 in the Supplementary Materials. The concentration of LMPs was expressed as ng g^−1^ dry weight (DW). The limit of quantity (LOQ) was calculated based on a signal/noise (S/N) ratio of 10 limit of detection (LOD), and they were as follows in Table 2.

## 4. Conclusions

In summary, this study is essential for understanding the distribution characteristics of LMPs in marine sediment sourced from a typical subtropical coastal area of DYB. Six toxin compounds (OA, DTX1, PTX2, homo-YTX, AZA2, and GYM) were detected in sediment samples from DYB. In particular it was the first time that homo-YTX was detected in sediments from subtropical area of China. Although the tendency of aquatic organisms to accumulate and concentrate lipophilic chemical contaminants from the environment is well known, the marine environmental fate and effects of LMPs are unclear. From this study, it seems that the sediment is a potential receptor of LMPs and contributes notably to regional pollution via an algae–water–sediment exchange. Therefore, the existence of LMPs in the sediment is an important potential source of toxins in benthic organisms and cannot be ignored. The targeted area of DYB is an inner bay with a relatively small spatial scale, and the physicochemical parameters of sediments may not show significant differences, which is also the reason that LMP concentrations may have a relatively strong correlation with some sediment parameters. However, further studies should focus on determining the fate of LMPs in the marine environment and the relationship between LMPs in the benthic organisms and sediments.

## Figures and Tables

**Table 1:** The vegetative cells or resting cysts of toxigenic algae producing lipophilic marine phycotoxins recorded in the sea area of the South China Sea.

Species	Toxin Profiles	Category	Producing Toxins	Location	Reference
*D. caudata* *D. acuminata* complex	okadaic acid/pectenotoxin	vegetative cell	OA, DTX1, PTX2, and PTX2sa	DYB	[17]
*P. lima*	okadaic acid	vegetative cell	OA and DTX1	DYB, Hainan Island	[29,30]
*P. reticulatum* *L. polyedrum* *G. spinifera*	yessotoxin	cyst	-	the coast of the SCS	[22,31]
*K. selliformis* *A. ostenfeldii*	gymnodimine	vegetative cell	GYM	Hongkong sea area	[17,28]
*A. poporum*	azaspiracids	cyst	AZA2 and AZA40	Guangxi sea area, SCS	[25,26]
*A. ostenfeldii*	spirolide	cyst	-	-	[28]

**Table 2:** Instrument parameters used in the analytical method for lipophilic marine toxins with HPLC-MS/MS under the mode of multiple reaction monitoring.

Toxin	ESI Polarity	Precursor Ion	Q1 m/z	Q3 m/z		DP	CE	LOD (ng g^−1^)	LOQ (ng g^−1^)
				1	2				
OA ^a^	ESI ^−^	[M − H]^−^	803.5	255.0		−150	−66	0.007	0.02
					112.9	−150	−92		
YTX ^a^	ESI ^−^	[M − 2H]^2−^	570.5	467.3		−130	−43	0.090	0.03
					396.2	−130	−48		
DTX1 ^a^	ESI ^−^	[M − H]^−^	817.5	255.1		−180	−64	0.010	0.03
					113.0	−180	−100		
homo-YTX ^a^	ESI ^−^	[M − 2H]^2−^	577.4	474.4		−130	−50	0.090	0.03
					403.4	−130	−50		
DTX2 ^b^	ESI ^−^	[M − H]^−^	803.5	255.2		−180	−64	0.010	0.03
					113.1	−180	−100		
AZA1 ^a^	ESI ^+^	[M + H]^+^	842.5	824.5		150	47	0.035	0.11
					806.3	150	54		
AZA2 ^a^	ESI ^+^	[M + H]^+^	856.5	838.5		150	47	0.080	0.03
					672.4	150	78		
AZA3 ^a^	ESI ^+^	[M + H]^+^	828.5	810.5		150	47	0.080	0.03
					658.4	150	78		
SPX1 ^a^	ESI ^+^	[M + H]^+^	692.5	444.3		153	51	0.020	0.06
					164.2	153	55		
GYM ^a^	ESI ^+^	[M + H]^+^	508.4	490.3		135	33	0.010	0.03
					162.3	135	49		
PTX2 ^a^	ESI^+^	[M + NH_4_]^+^	876.5	823.4		150	36	0.060	0.02
					213.1	150	44		

Note: ^a^ stands for certified reference toxins; ^b^ stands for no certified reference toxin.

## Supplementary Materials

The following are available online at https://www.mdpi.com/1660-3397/17/11/623/s1. Figure S1. The linear relationship between the total toxin concentration and main parameters of sediment from Daya Bay (*p* > 0.05, no significant difference; *p* < 0.05, significant difference). Figure S2. Selected LC-MS/MS chromatograms for standards of lipophilic marine phycotoxins (left) and sediment samples of Daya Bay (right). Figure S3. The standard work curve of lipophilic marine toxins. Table S1. Concentration of multiple lipophilic marine phycotoxins in surface sediments sourced from Daya Bay in different sampling times from 2015 to 2017. Table S2. The granulometric parameters of surficial sediments sourced from Daya Bay in different sampling time from 2015 to 2016. Table S3. TN, TOC, C/N, Eh and temperature of surficial sediments sourced from Daya Bay in different sampling time from 2015 to 2016.
