# Supplementary material for: Sediment as a Potential Pool for Lipophilic Marine Phycotoxins with the Case Study of Daya Bay of China
Source: Mar Drugs. 2019 Oct 31;17(11):623. doi: 10.3390/md17110623 (PMC6891265; doi:10.3390/md17110623)
Supplement: Supplementary file 1 [file marinedrugs-17-00623-s001.pdf]

Supplementary Materials: Sediment as a potential pool for lipophilic marine phycotoxins with the case study of Daya Bay of China

Yang Liu[1, 3], Sen Du[1, 3, 4], Peng Zhang[1, 3, 4], Zhuoru Lin[2, 4, 5], Yanyan Zhou[1, 3], Lizhao, Chen[1, 3], Rencheng Yu[2, 5*], Zhang Li[1, 3*]

1 Key Laboratory of Tropical Marine Bio-resources and Ecology, Guangdong Provincial Key Laboratory of Applied Marine Biology, South China Sea Institute of Oceanology, Chinese Academy of Sciences, Guangzhou, 510301, China; E-Mail: liuyang@scsio.ac.cn; zhangpeng_nanjing@126.com; dusen14@mails.ucas.ac.cn; zhouyyahgy@scsio.ac.cn; chenlizhao13@mails.ucas.ac.cn; zhangli@scsio.ac.cn.

2 Laboratory for Marine Ecology and Environmental Science, Qingdao National Laboratory for Marine Science and Technology;

3 Southern Marine Science and Engineering Guangdong Laboratory (Guangzhou);

4 Institution of South China Sea Ecology and Environmental Engineering, Chinese Academy of Sciences; E-Mail: linzhuoru@outlook.com; rcyu@qdio.ac.cn;

5 University of Chinese Academy of Sciences, Beijing 100039

* Author to whom correspondence should be addressed. Tel.: +86 532 82898590; Fax: +86 532 82898590 (R.C. Yu) and Tel: +86 020 89221322; Fax: +86 020 89221322 (L. Zhang).

Table Captions:

Table S1. Concentration of multiple lipophilic marine phycotoxins in surface sediments sourced from Daya Bay in different sampling times from 2015 to 2017.

Table S2. The granulometric parameters of surficial sediments sourced from Daya Bay in different sampling time from 2015 to 2016.

Table S3. TN, TOC, C/N, Eh and temperature of surficial sediments sourced from Daya Bay in different sampling time from 2015 to 2016.

Table S1. Concentration of multiple lipophilic marine phycotoxins in surface sediments sourced from Daya Bay in different sampling times from 2015 to 2017.

Location	Toxin profiles (ng/g)										longitude	latitude
	OA	YTX	DTX1	homo-YTX	AZA1	AZA2	AZA3	SPX1	GYM	PTX2		
August 2015												
S1	0.34	0.00	0.73	0.23	0.00	0.14	0.00	0.00	0.00	0.07	114.74	22.76
S3	0.00	0.00	0.00	0.00	0.00	0.00	0.00	0.00	0.07	0.00	114.59	22.72
S5	3.94	0.00	4.96	0.35	0.00	0.13	0.00	0.00	0.00	0.14	114.67	22.68
March 2016												
S1	0.30	0.00	0.00	0.00	0.00	0.22	0.00	0.00	0.02	0.14	114.74	22.76
S2	0.35	0.00	0.73	0.17	0.00	0.10	0.00	0.00	0.15	0.16	114.64	22.73
S3	0.00	0.00	0.39	0.00	0.00	0.00	0.00	0.00	0.06	0.05	114.59	22.72
S4	0.00	0.00	0.00	0.00	0.00	0.22	0.00	0.00	0.00	0.12	114.72	22.70
S5	0.22	0.00	0.00	0.00	0.00	0.03	0.00	0.00	0.02	0.25	114.67	22.68
S6	0.00	0.00	0.00	0.00	0.00	0.04	0.00	0.00	0.05	0.03	114.62	22.68
S7	0.00	0.00	0.00	0.00	0.00	0.10	0.00	0.00	0.00	0.11	114.55	22.68
S8	0.00	0.00	0.00	0.00	0.00	0.00	0.00	0.00	0.01	0.13	114.72	22.61
S8	0.00	0.00	0.00	0.00	0.00	0.02	0.00	0.00	0.01	0.22	114.72	22.61
S9	0.15	0.00	0.20	0.07	0.00	0.03	0.00	0.00	0.04	0.25	114.68	22.61
S10	0.18	0.00	0.37	0.16	0.00	0.19	0.00	0.00	0.02	0.21	114.62	22.60
S11	0.00	0.00	0.00	0.00	0.00	0.07	0.00	0.00	0.02	0.22	114.57	22.61
S12	0.13	0.00	0.28	0.06	0.00	0.10	0.00	0.00	0.02	0.18	114.52	22.57
S13	0.12	0.00	0.22	0.00	0.00	0.05	0.00	0.00	0.02	0.23	114.76	22.53
S14	0.00	0.00	0.00	0.00	0.00	0.10	0.00	0.00	0.00	0.09	114.64	22.53
November 2016												
S1	0.20	0.00	0.39	0.30	0.00	0.10	0.00	0.00	0.00	0.07	114.74	22.76
S2	0.10	0.00	0.29	0.00	0.00	0.02	0.00	0.00	0.00	0.11	114.64	22.73
S3	0.15	0.00	0.11	0.12	0.00	0.03	0.00	0.00	0.05	0.04	114.59	22.72
S4	0.25	0.00	0.40	0.17	0.00	0.18	0.00	0.00	0.00	0.14	114.72	22.70
S5	0.05	0.00	0.00	0.00	0.00	0.00	0.00	0.00	0.01	0.56	114.67	22.68
S6	0.38	0.00	0.21	0.16	0.00	0.14	0.00	0.00	0.03	0.10	114.62	22.68
S7	0.72	0.00	0.91	0.07	0.00	0.09	0.00	0.00	0.00	0.08	114.55	22.68
S8	0.11	0.00	0.13	0.00	0.00	0.03	0.00	0.00	0.01	0.27	114.72	22.61
S9	0.65	0.00	1.09	0.06	0.00	0.05	0.00	0.00	0.05	0.33	114.68	22.61
S10	0.22	0.00	0.16	0.08	0.00	0.16	0.00	0.00	0.02	0.17	114.62	22.60
S11	0.00	0.00	0.17	0.12	0.00	0.05	0.00	0.00	0.00	0.24	114.57	22.61
S12	0.00	0.00	0.00	0.00	0.00	0.05	0.00	0.00	0.00	0.04	114.52	22.57
S13	0.00	0.00	0.00	0.00	0.00	0.02	0.00	0.00	0.00	0.11	114.76	22.53
S14	1.34	0.00	2.13	0.06	0.00	0.09	0.00	0.00	0.02	0.21	114.64	22.53
August 2017												
S4	0.00	0.00	0.00	0.00	0.00	0.05	0.00	0.00	0.00	0.10	114.72	22.70
S4	0.00	0.00	0.13	0.00	0.00	0.01	0.00	0.00	0.00	0.24	114.72	22.70

S8	0.00	0.00	0.00	0.00	0.00	0.00	0.00	0.00	0.00	0.06	114.72	22.61
S9	0.00	0.00	0.00	0.00	0.00	0.08	0.00	0.00	0.00	0.06	114.68	22.61
S10	0.00	0.00	0.61	0.00	0.00	0.01	0.00	0.00	0.00	0.14	114.62	22.60
S11	0.00	0.00	0.33	0.00	0.00	0.00	0.00	0.00	0.00	0.07	114.57	22.61
S12	0.00	0.00	0.14	0.00	0.00	0.04	0.00	0.00	0.00	0.01	114.52	22.57
S13	0.16	0.00	0.00	0.00	0.00	0.02	0.00	0.00	0.00	0.07	114.76	22.53
S14	0.00	0.00	0.00	0.06	0.00	0.10	0.00	0.00	0.00	0.14	114.64	22.53

Table S2. The granulometric parameters of surficial sediments sourced from Daya Bay in different sampling time from 2015 to 2016.

Location	sand（%）			silt (%)			clay (%)			mean grain size			median diameter		
	Jul. 2015	Mar. 2016	Nov. 2016	Jul. 2015	Mar. 2016	Nov. 2016	Jul. 2015	Mar. 2016	Nov. 2016	Jul. 2015	Mar. 2016	Nov. 2016	Jul. 2015	Mar. 2016	Nov. 2016
S1	0.6	0.7	28.6	61.6	60.9	48.4	37.7	38.4	11.1	7.6	7.7	4.8	7.5	7.5	5.2
S2	1.3	2.5	58.5	60.4	60.5	20.3	38.3	37.1	2.9	7.7	7.6	2.0	7.5	7.5	2.8
S3	0.8	0.8	0.5	66.8	65.6	63.1	32.5	33.5	36.5	7.4	7.4	7.5	7.3	7.3	7.4
S4	0.6	0.5	0.1	65.0	64.4	62.4	34.4	35.0	37.5	7.5	7.5	7.6	7.4	7.4	7.5
S5	5.8	28.0	44.5	63.0	48.8	30.0	31.1	23.2	6.7	7.1	6.0	2.5	7.1	6.3	3.2
S6	1.6	0.6	0.7	59.8	64.1	62.5	38.6	35.3	36.8	7.6	7.5	7.6	7.5	7.4	7.5
S7	0.2	0.5	0.7	62.7	62.5	59.1	37.1	37.0	40.2	7.6	7.6	7.7	7.5	7.5	7.6
S8	47.5	48.8	41.0	33.3	33.0	34.5	19.2	18.2	24.6	5.1	5.0	5.8	4.3	4.1	6.1
S9	34.3	10.1	41.6	42.1	60.7	38.3	23.6	29.1	20.1	5.9	7.0	5.4	6.4	7.1	5.1
S10	0.3	1.6	0.6	65.5	66.2	65.1	34.1	32.2	34.4	7.5	7.4	7.5	7.4	7.3	7.4
S11	14.4	22.7	20.6	68.1	62.0	62.6	17.5	15.4	16.8	5.8	5.5	5.7	5.3	4.9	5.0
S12	11.4	5.9	4.6	64.9	67.3	66.5	23.6	26.8	28.9	6.4	6.7	6.8	6.3	6.8	6.9
S13	8.1	10.5	8.8	66.6	66.6	66.2	25.4	22.9	25.0	6.7	6.5	6.6	6.7	6.5	6.6
S14	3.3	4.1	2.9	67.7	67.2	69.0	28.9	28.6	28.1	7.0	7.0	7.0	6.9	6.9	7.0
mean	9.3	9.8	18.1	60.5	60.7	53.4	30.1	29.5	25.0	6.9	6.9	6.0	6.8	6.8	6.1
STDEV	14.3	14.2	20.7	10.2	9.3	16.0	7.2	7.3	12.0	0.8	0.8	1.9	1.0	1.0	1.6

Table S3. TN, TOC, C/N, Eh and temperature of surficial sediments sourced from Daya Bay in different sampling time from 2015 to 2016.

Location	TON（%）			TOC (%)			C/N			Eh (mV)			Temperature (℃)		
	Jul. 2015	Mar. 2016	Nov. 2016	Jul. 2015	Mar. 2016	Nov. 2016	Jul. 2015	Mar. 2016	Nov. 2016	Jul. 2015	Mar. 2016	Nov. 2016	Jul. 2015	Mar. 2016	Nov. 2016
S1	0.2	0.2	0.2	1.3	1.5	1.5	6.1	6.8	8.1	-267.0	-133.0	-250.0	29.8	16.7	27.3
S2	0.1	0.2	0.1	0.9	1.1	0.9	6.4	6.6	12.1	-170.0	-212.0	-255.0	30.3	16.5	27.3
S3	0.2	0.1	0.1	1.0	1.1	1.4	6.3	7.7	13.1	-144.0	-114.0	-319.0	30.2	16.2	26.9
S4	0.2	0.2	0.2	1.2	1.5	1.3	6.4	6.6	7.2	-196.0	-207.0	-307.0	28.2	16.5	27.0
S5	0.1	0.1	0.1	0.8	0.8	1.1	6.7	10.7	11.2	-161.0	-97.0	-145.0	29.6	16.2	27.0
S6	0.2	0.2	0.2	1.2	1.4	1.6	5.7	6.7	10.0	-270.0	-182.0	-324.0	28.1	16.4	27.4
S7	0.2	0.2	0.2	1.2	1.7	1.5	6.4	6.9	8.2	-187.0	-192.0	-309.0	29.4	16.4	27.0
S8	0.0	0.1	0.0	0.4	0.5	0.6	9.3	7.3	12.4	-129.0	-129.0	-120.0	26.5	16.3	27.3
S9	0.1	0.1	0.0	0.6	1.0	0.4	6.7	6.4	11.3	-201.0	-139.0	-193.0	26.6	16.1	27.2
S10	0.2	0.2	0.2	1.1	1.2	1.2	6.3	6.4	7.6	-109.0	-104.0	-232.0	25.9	16.0	27.1
S11	0.1	0.1	0.1	0.6	0.6	0.5	7.1	6.2	9.3	-139.0	-87.0	-232.0	27.6	16.0	27.2
S12	0.1	0.2	0.2	0.8	1.7	2.0	6.7	9.9	12.7	-124.0	-123.0	-258.0	27.3	17.1	27.7
S13	0.1	0.1	0.1	0.7	0.7	0.7	6.0	6.3	9.5	-129.0	-164.0	-211.0	24.7	15.8	27.0
S14	0.2	0.1	0.1	0.9	1.0	1.0	5.1	6.9	11.0	-153.0	-99.0	-269.0	26.1	15.8	26.6
mean	0.1	0.2	0.1	0.9	1.1	1.1	6.5	7.2	10.3	-169.9	-141.6	-244.6	27.9	16.3	27.1
STDEV	0.1	0.1	0.1	0.3	0.4	0.5	0.9	1.4	2.0	50.0	42.5	62.2	1.8	0.4	0.3

Figure Captions:

Figure S1. The linear relationship between the total toxin concentration and main parameters of sediment from Daya Bay ($P > 0.05$, no significant difference; $P < 0.05$, significant difference).

Figure S2. Selected LC-MS/MS chromatograms for standards of lipophilic marine phycotoxins (left) and sediment samples of Daya Bay (right).

Figure S3. The standard work curve of lipophilic marine toxins.

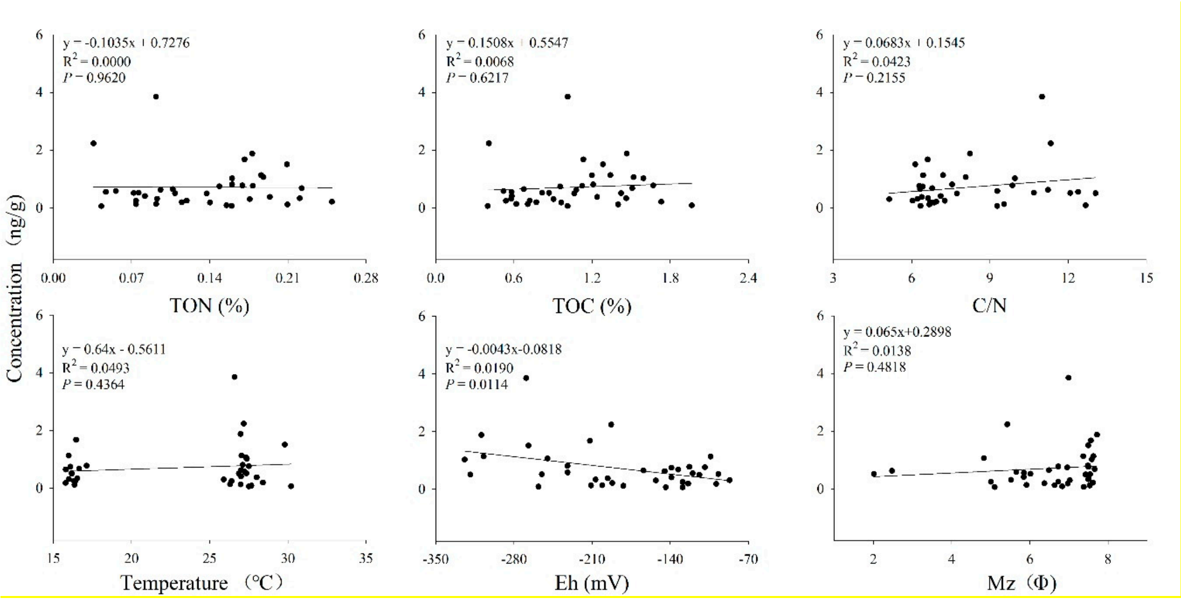

Figure S1. The linear relationship between the total toxin concentration and main parameters of sediment from Daya Bay (*P* > 0.05, no significant difference; *P* < 0.05, significant difference).

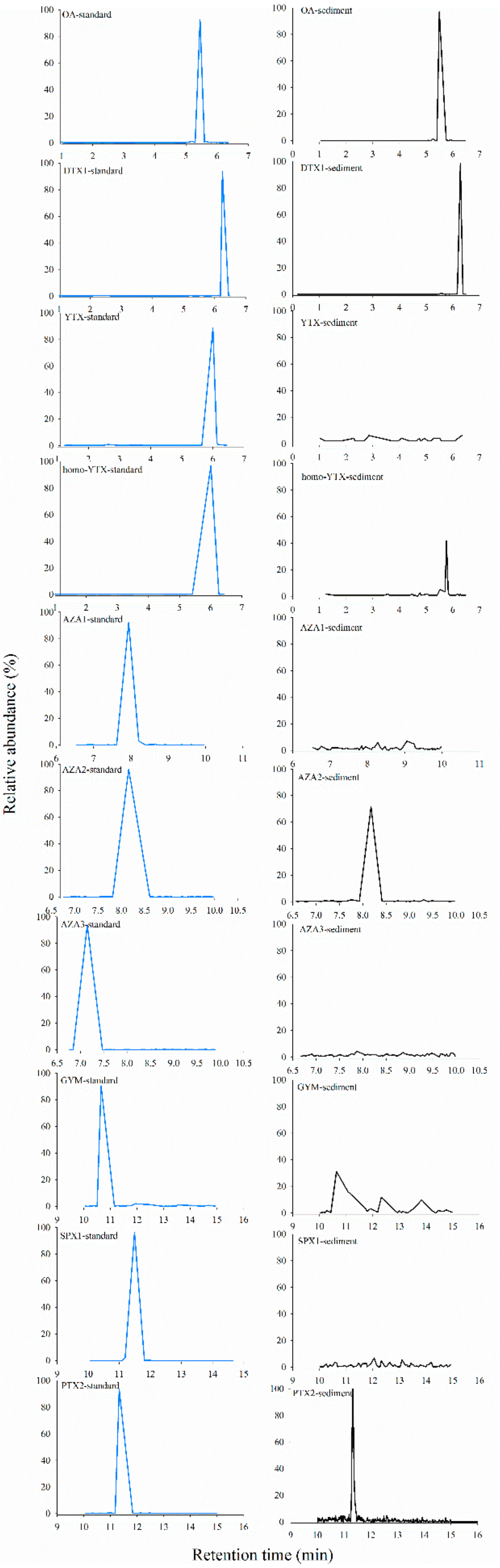

Figure S2. Selected LC-MS/MS chromatograms for standards of lipophilic marine phycotoxins (left) and sediment samples of Daya Bay (right).

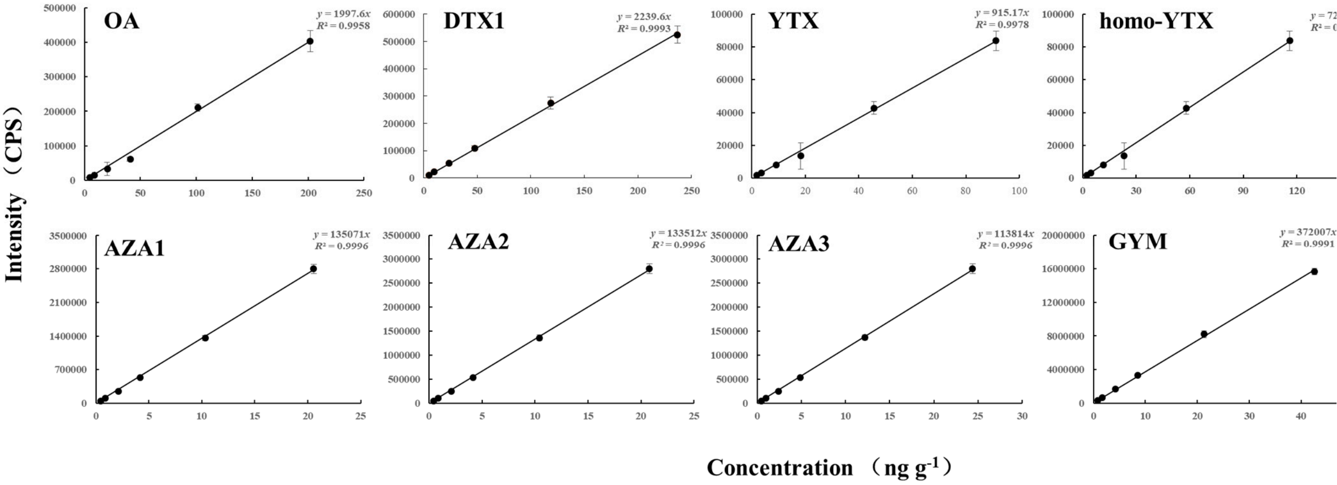

Figure S3. The standard work curve of lipophilic marine toxins.
